# Function of Non-coding RNA in *Helicobacter pylori*-Infected Gastric Cancer

**DOI:** 10.3389/fmolb.2021.649105

**Published:** 2021-05-11

**Authors:** Chao Wang, Yiyang Hu, Huan Yang, Sumin Wang, Bo Zhou, Yulu Bao, Yu Huang, Qiang Luo, Chuan Yang, Xia Xie, Shiming Yang

**Affiliations:** Department of Gastroenterology, Xinqiao Hospital, Third Military Medical University, Chongqing, China

**Keywords:** non-coding RNA, *Helicobacter pylori* infection, gastric cancer, genetic polymorphisms, chemoresistance

## Abstract

Gastric cancer is a common malignant tumor of the digestive system. Its occurrence and development are the result of a combination of genetic, environmental, and microbial factors. *Helicobacter pylori* infection is a chronic infection that is closely related to the occurrence of gastric tumorigenesis. Non-coding RNA has been demonstrated to play a very important role in the organism, exerting a prominent role in the carcinogenesis, proliferation, apoptosis, invasion, metastasis, and chemoresistance of tumor progression. *H. pylori* infection affects the expression of non-coding RNA at multiple levels such as genetic polymorphisms and signaling pathways, thereby promoting or inhibiting tumor progression or chemoresistance. This paper mainly introduces the relationship between *H. pylori*-infected gastric cancer and non-coding RNA, providing a new perspective for gastric cancer treatment.

## Introduction

As one of the most common digestive tumors worldwide, the morbidity and mortality of gastric cancer (GC) are increasing annually (Ferlay et al., 2019). The associated risk factors include smoking, overweight, salty food consumption, Epstein–Barr virus infection, and exposure to asbestos. Surgery, chemotherapy, and chemoradiation are the main treatments for GC, but the prognosis is not satisfactory. Numerous studies have shown that the pathogenesis and progression of GC are closely related to those of *Helicobacter pylori* infection (Helicobacter and Cancer Collaborative Group [HCCG], 2001). *H. pylori* is regarded as a gram-negative microaerophilic bacterium that is capable of entering the human body early and colonizing the mucosal area of the stomach for a long time (Blaser and Atherton, 2004). *H. pylori* infection is associated with inducing chronic gastritis, peptic ulcer, GC, and mucosa-associated lymphoid tissue (MALT) lymphoma (Petra et al., 2017). Various clinical analysis and basic biological research have revealed that patients with *H. pylori*-positive GC have more lymph node metastasis and a worse prognosis than have negative patients. Therapy for *H. pylori* eradication can effectively prevent GC (Choi et al., 2018; Mera et al., 2018; Suzuki and Matsuzaki, 2018). The Kyoto Global Consensus Report recommends that regardless of age or severity of gastric mucosal lesions, especially in areas with a high incidence of GC, all *H. pylori*-infected patients should be treated (Sugano et al., 2015; Malfertheiner et al., 2017; Sugano, 2019).

Non-coding RNA (ncRNA) refers to RNA that does not encode protein, which has been divided into long ncRNAs (lncRNAs) and short ncRNAs including microRNAs (miRNAs), PiWi-interacting RNAs (piRNAs), small nucleolar RNAs (snoRNAs), small interfering RNAs (siRNAs), tRNA-derived small RNAs (tsRNAs), circular RNAs (circRNAs), and heterochromatin-derived 24nt small RNA in plants according to their length. These RNAs are derived from genomic transcription, but they are not translated into proteins; and they play their respective biological roles at the RNA level. Among these RNAs, lncRNA, miRNA, and some special small ncRNAs (sncRNAs) are mainly involved in the progress of *H. pylori*-induced GC. LncRNA is an ncRNA that is greater than 200 nucleotides in length. It has many known functions, including transcriptional interference, regulation of alternative splicing, generation of endogenous siRNA, regulation of protein activity, and alteration of protein positioning (Wilusz et al., 2009). In addition, many studies have shown that lncRNA is more tissue-specific than mRNA, indicating that it is also closely related to the function of the tissue (Ransohoff et al., 2018). MiRNA is a non-coding 18- to 24-nucleotide RNA that regulates gene expression at the mRNA level. Mature miRNA can directly bind to the 3′UTR region of the target gene to rapidly degrade mRNA or inhibit protein expression (Bartel, 2004; Acunzo et al., 2015; Shomali et al., 2017).

This review mainly summarizes the mechanism of ncRNA in *H. pylori*-infected GC. *H. pylori* infection modulates expression of ncRNA and changes the expression of related target genes. Their impact on tumor progression and drug resistance treatment has been categorized and summarized, and a new perspective for clinical treatment is provided.

## *Helicobacter Pylori* Plays a Vital Role in Gastric Cancer

The prevalence of *Helicobacter pylori* presents large regional differences worldwide, which is related to factors such as geography and basic health conditions. *H. pylori* survival is facilitated in an acidic environment, and it colonizes in the gastric mucosa by virtue of its spiral shape, exercise ability, adhesion factors, and urease and ammonia production, subsequently producing a complex inflammatory response, damaging the gastric mucosa, and subsequently producing digestive diseases via the expression of various pathogenic markers such as cytotoxin-related gene A (CagA), BabA adhesin, and empty vesicular toxin (VacA) (Backert et al., 2017). Flagellar movement and various adhesion factors (AlpA/B, BabA, OipA, SabA, and HopQ) promote *H. pylori* adhesion to epithelial cells. Urease converts urea into ammonia, making the environment in which bacteria live weakly acidic and thereby reducing the level of intestinal bacteria. VacA produces proteins that are toxic to gastrointestinal epithelial cells (Morello, 1999; Amieva and El-Omar, 2008; Atherton and Blaser, 2009; Safaralizadeh et al., 2017; Su et al., 2019). The virulence factor CagA is involved in various signal transduction processes (Pachathundikandi et al., 2013). All these determine the importance of *H. pylori* in GC.

## Non-Coding RNA Influences the Progression and Treatment of *Helicobacter Pylori*-Infected Gastric Cancer

### Small RNA

Small ncRNAs produced by bacteria are classified as sRNAs, which exert their heterogeneity in a eubacterial environment. The length of sRNA ranges from 50 to 250 nucleotides, and its effect on biological process and its target genes have been identified by various methods *in vitro* and *in vivo* (Vogel and Wagner, 2007; Sharma and Vogel, 2009). The present mechanism displays a binding function to protein or an antisense RNA role on *trans*-encoded mRNAs, in which the latter usually shows translation inhibition or activation through imperfect complementarity between sRNA and its targets, modulating the stability and/or accessibility on the translational machinery (Majdalani et al., 2005; Livny and Waldor, 2007). The sRNAs have also been reported to participate in acid resistance in *Escherichia coli* (Opdyke et al., 2004; Tramonti et al., 2008), virulence of pathogens (Geissmann et al., 2006; Romby et al., 2006), and iron homeostasis (Chen and Crosa, 1996; Dühring et al., 2006). *Helicobacter pylori* also produced sRNAs that participate in the progression of GC. A large number of sRNAs were found in an analysis of *H. pylori* primary transcriptome study (Rieder et al., 2012). Reports show that bacterial Sm-like protein Hfq is necessary for effective function of sRNA (Valentin-Hansen et al., 2004). However, Hfq, an RNA molecular chaperone, is absent in *H. pylori*. By facilitating the pairing of small RNAs with their target mRNAs, Hfq can affect translation and turnover rates of specific transcripts and contribute to complex posttranscriptional networks (Vogel and Luisi, 2011). Thus, *H. pylori* was previously thought to lack ribosomal regulation (Mitarai et al., 2007). Because of the lack of Hfq in *H. pylori*, two methods were designed to identify other auxiliary proteins in sRNA-mediated regulation, and RNA–protein interactions were identified between ribosomal protein S1 and various mRNA and sRNA of *H. pylori*, which confirmed that *H. pylori* can control their gene expression via ribosomal regulation (Rieder et al., 2012). The identification of *H. pylori* sRNA and the mechanism of ribose regulation have potential effects on the virulence mechanism and stress response (Pernitzsch and Sharma, 2012), which may be associated with the development of GC.

The HP0165–HP0166 two-component system (TCS) in *H. pylori* participates in the increased expression of urease genes, while whether the increased activity of urease is beneficial or harmful to organism is determined by the presence or absence of acid in the stomach (Clyne et al., 1995; Meyer-Rosberg et al., 1996; Pflock et al., 2004, 2005). HP0165 is the membrane sensor, while HP0166 is its response regulator. For survival and colonization on the gastric surface, *H. pylori* regulated TCS to change urease activity according to the different intragastric pH values (Wen et al., 2006). A novel *cis*-encoded antisense sRNA, identified as 5′ureB-sRNA, downregulates ureAB expression by enhancing transcription termination of 5′ region of ureB, which validates through an *in vitro* transcription assay (Wen et al., 2013). However, HP0165–HP0166 TCS negatively regulates expression of 5′ureB-sRNA to increase ureAB expression at low pH values and enhances 5′ureB-sRNA to decrease ureAB expression and to decrease urease activity at high pH values (Wen et al., 2011).

CncR1, a rich and conserved sRNA encoded by the virulence-associated cag pathogenicity island (cag-PAI) of *H. pylori*, interacts with the fliK mRNA and downregulates bacterial motility and adhesion ability, significantly impairing bacterial adhesion to host gastric cell lines (Vannini et al., 2016). The sRNA RepG (Regulator of polymeric G-repeats) in *H. pylori* was also found to directly target a variable homopolymeric G-repeat in the leader of the TlpB chemotaxis receptor mRNA, which contains simple sequence repeats (SSRs) (Pernitzsch et al., 2014). Phase variation in hypermutable SSRs contributes to host adaptation of bacterial pathogens. In this way, sRNA may ensure the survival of *H. pylori* in the human body, leading to severe disease. In fact, many features of the sRNA are unknown and worth exploring, as they may have important implications for *H. pylori*-induced GC.

**FIGURE 1:**
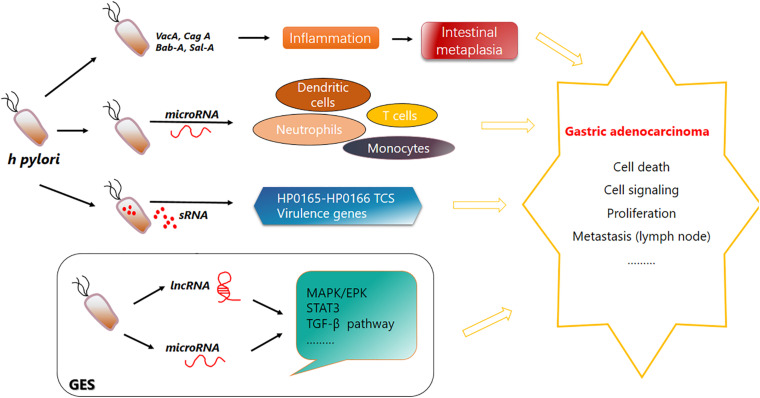
Function of *Helicobacter pylori* in gastric carcinogenesis. *H. pylori* secretes Bab-A, Sab-A, VacA, CagA, and other substances to help invade and colonize in the human gastric mucosa and cause chronic inflammation and superficial gastritis. Its infection leads to a variety of signaling events. Immune cells such as dendritic cells, neutrophils, monocytes, and T cells can produce various microRNAs. Specific sRNA can combine with the HP0165–HP0166 two-component system to regulate the pH adaptability of *H. pylori* to help its inhabitation and colonization in stomach. *H. pylori* invasion also causes upregulation or downregulation of lncRNAs and microRNAs. Their complex regulatory network is disrupted, and pathways are affected. Bab-A, blood-group antigen binding adhesion; Sab-A, sialic acid binding protein A.

*Helicobacter pylori* not only plays a role via its own nosotoxin but also participates in the homeostasis regulation of intestinal flora, and *H. pylori* eradication treatment has a significant effect on the change in intestinal flora (Zhang et al., 2016). For example, after the application of PPI for *H. pylori* eradication treatment, the abundance of streptococci, enterococci, staphylococci, and micrococci in the intestinal flora increased while clostridia decreased (Freedberg et al., 2015). A schematic diagram of the pathogenic mechanism of *H. pylori* is shown (Figure 1).

### Long Non-coding RNA

#### The Mechanism of Long Non-coding RNAs in *Helicobacter pylori-*Infected Gastric Cancer

Polymorphisms in lncRNAs have been reported to influence the splicing and stability of mRNA (Burd et al., 2010; Chung et al., 2011), and the special region known as the “gene desert” was discovered to participate in prostate cancer, colorectal cancer, breast cancer, and *H. pylori*-infected GC (Tomlinson et al., 2007; Ghoussaini et al., 2008; Zhang et al., 2010; He et al., 2017). A case–control study revealed that rs16901946 of prostate cancer ncRNA 1 (PRNCR1) was associated with GC risk, which could be increased by *H. pylori* infection. Another case–control study showed that LINC00673 rs11655237 with GG genotype was more susceptible to *H. pylori* infection.

Based on the database analysis of The Cancer Genome Atlas (TCGA), a competing endogenous RNA (ceRNA) network was constructed, and four lncRNAs (LINC01254, LINC01287, LINC01524, and U95743.1) showed higher expression in *H. pylori* infection-positive GC patients, and the results were validated by real-time PCR. In contrast, microarray analysis showed different results, in which 23 lncRNAs were upregulated and 21 downregulated [such as lncRNA NR_026827 (Zhong et al., 2018)] in *H. pylori*-infected GES-1 gastric epithelial cell, and some of the results were also found by PCR (Yang et al., 2015; Polakovicova et al., 2018). Among them, a recent network analysis showed that RP11-169F17.1 and RP11-669N7.2 were related to *H. pylori* infection-induced gastritis, as their targets showed great overlap with *H. pylori*-infection associated genes (Yang and Song, 2019). Seven upregulated and 17 downregulated lncRNAs were found using GSE5081 and GSE66229 (Zhang et al., 2020). LINC00152 and H19 were previously shown to be significantly upregulated in GC patients’ blood samples and cancer tissues, which was validated to be a risk factor in the diagnosis and prognosis of GC with *H. pylori* infection (Pang et al., 2014; Yang T. et al., 2016).

Regarding the underlying mechanism, the lncRNA–RNA network was constructed based on the profiling of *H. pylori*-infected GES-1 cells, for which a concrete example is the downregulation of MUC2 by lncRNA AF147447 to suppress cell proliferation and invasion in the *H. pylori*-infection background (Zhu et al., 2015; Zhou et al., 2016). According to the RNA-seq analysis of *H. pylori*-infected AGS cells, THAP domain-containing nine antisense RNA 1 (THAP9-AS1) was found to be induced by *H. pylori* infection and to promote the migration and proliferation of GC cells (Jia et al., 2019). The expression level of lnc-SGK1 was elevated as a consequence of *H. pylori* infection and a high-salt diet. Lnc-SGK1 increases the transcription of SGK1 in a *cis*-regulatory manner, which activates JunB and disrupts T helper cell differentiation (Yao et al., 2016). The results of lncRNA were mainly derived from database analysis, and there was a lack of clinical studies.

#### Long Non-coding RNAs Influence the Drug Resistance and Prognosis of *Helicobacter pylori*-Infected Gastric Cancer

Chemotherapy can achieve a certain effect on the treatment of GC, but the acquisition of drug resistance will lead to the failure of chemotherapy in GC patients. Although the mechanism of anticancer drug resistance has been extensively studied, its specific mechanism has not yet been elucidated. In recent years, more and more studies have shown that ncRNA plays a regulatory role in the generation and maintenance of drug resistance. Cisplatin is a commonly used drug in the treatment of GC (Zheng et al., 2017), but its resistance has been found to be closely related to ncRNA. It is reported that lncRNA BCAR4 expression was enhanced in cisplatin-resistant cell strain SGC7901/DDP. And the drug resistance of cell strains was positively correlated with the expression level of BCAR4 (Wang L. et al., 2017). And it was found that the removal of lncRNA ANRIL inhibited the development of multidrug resistance (MDR) in GC cells (Lan et al., 2016). At present, many ncRNAs have been found to play a regulatory role in chemotherapy resistance of GC, and even some ncRNAs play a relatively key role. Therefore, ncRNAs can be used to be a kind of candidate drugs to develop new molecular targeted therapy strategies or reverse the resistance of GC cells to chemotherapy.

Besides, lncRNA is also related to prognosis of GC. Researchers found that the knockdown of lncRNA CASC19 inhibited proliferation and migration of GC cells *in vitro*. And their multivariate Cox analysis confirmed that CASC19 overexpression was an independent prognostic factor for overall survival (Wang et al., 2019). A set of 24-lncRNAs significantly associated with disease-free survival (DFS) was even established and used to improve prognosis prediction of GC (Zhu et al., 2016). Actually, miRNAs have been studied for much longer than lncRNAs. And at the same time, there are more research results.

### MicroRNA

#### The Mechanism of MicroRNAs in *Helicobacter pylori*-Infected Gastric Cancer

From the perspective of polymorphism, miR-27a rs895819 and *H. pylori* have shown an interaction effect in gastric carcinogenesis (Xu et al., 2017). In contrast, miR-124a, miR-34b, and miR-34c have been reported to be downregulated in *H. pylori*-infected gastric mucosa, and miR-124a downregulation is associated with CpG hypermethylation of the miR-124a3 locus and higher IL-8 expression (Tahara et al., 2019).Additionally, miR-124 downregulation leads to elevated expression of spermine oxidase (SMOX) as it directly binds directly to the 3′UTR of SMOX mRNA, and this process can be reversed by 5-azacytidine (Murray-Stewart et al., 2016). *H. pylori* eradication induces decreased methylation (*p* < 0.01) and increased expression (*p* = 0.03) of miR-133a (Hyun Lim et al., 2018). MiR-204 is upregulated in GC compared with *H. pylori*-positive gastritis (*p* < 0.004) (Kuo et al., 2019). Other examples about phenotype changes associated with miRNA in *H. pylori-*infected GC have been mentioned in Table 1.

**TABLE 1:** Associated phenotype alteration in non-coding RNA.

MicroRNA	Phenotype change	References
MiR-27	rs895819	Xu et al., 2017
MiR-124a	CpG hypermethylation	Tahara et al., 2019
MiR-129-2	Methylation	Watari et al., 2019
MiR-133a	Methylation	Hyun Lim et al., 2018
MiR-149	Hypermethylation	Li et al., 2015
MiR-200a/b	Methylation	Choi et al., 2020
MiR-204(TRPM3)	Methylation	Chen et al., 2019
MiR-210	Methylation	Kiga et al., 2014
MiR-490-3p(CHRM2)	Methylation	Shen et al., 2015; Cho et al., 2016
MiR-4795	rs1002765	Wu et al., 2017
let-7b	rs8111742	Isomoto et al., 2012; Hayashi et al., 2013; Wu et al., 2017
lncPRNCR1	rs16901946	He et al., 2017
LINC00673	rs11655237	Zhao et al., 2019

Based on the mechanism, the change in miRNA expression is closely related to *H. pylori*-produced virulence factors. MiR-34a was found to be significantly reduced in the *H. pylori* + GC group by rTip-α (a toxin secreted by *H. pylori*), while its overexpression decreased the level of TLP4, TNF-α, and IL-6. Viability was enhanced by rTip-α but decreased by miR-34a, which induces cell proliferation (Wang et al., 2018). Lipopolysaccharide (LPS) from *H. pylori* activates sp1 to increase MDM2 expression, while MDM2 represses p63 to inhibit Dicer, leading to inhibition of miR-106b and miR-375. JAK1 and STAT3 are downstream target genes of miR-106b (Ye et al., 2015). MiR-134 targets FoxM1 (Forkhead box protein M1) to suppress the proliferation, invasion, and epithelial–mesenchymal transition (EMT) of GC cell, while *H. pylori*_CagA+/P+_ [CagA and penicillin-binding protein 1A (PBP1A) mutation-positive] infection suppresses miR-134 expression when compared with *H. pylori*_CagA+/P–_ tissues (Huang et al., 2019). The miR-155 was found to be upregulated by CagA (cytotoxin-associated gene A) from *H. pylori*, and it can restrict KLF4 (Krüppel-like transcription factor) expression to promote EMT and tumor growth (Ou et al., 2019).

Many signaling pathways are involved in the regulation between *H. pylori* infection and miRNA. *H. pylori* infection causes activation of the NF-κB signaling pathway, which leads to miR-7 downregulation, while miR-7 targets the IκB kinase IKKε to repress RELA activation. In return IKKε and RELA repress miR-7. Thus, the repression of RELA and FOS is released, and cell proliferation and tumorigenesis are promoted (Zhao et al., 2015). *H. pylori* infection activates NF-κB, increases IL-6 secretion, and promotes AP-1 and STAT3, which induce transcription of miR-21; and it plays an oncogenic role in cancer development, including proliferation, migration, and apoptosis (Zhang et al., 2008; Belair et al., 2009; Ma and Tao, 2012). MiR-21 activates COX2, which participates in preneoplastic gastric lesions that are resistant to apoptosis (Shukla et al., 2016). MiR-3178 decreases the expression of TRAF3, TNF-α, and IL-6, accompanied by the inhibition of NF-κB signals, while *H. pylori* infection presents Tip-α to inhibit miR-3178 expression, thus activating the NF-κB signal and promoting inflammation and carcinogenesis (Zou et al., 2017). NF-κB is also involved in the upregulation of miR-223-3p by binding to the promoter of primary miR-223-3p. *H. pylori* infection promotes FZD7 (Frizzled 7) expression, which is an important coreceptor in the WNT signaling pathway, promoting cell proliferation, while miR-27b targets the 3′UTR of FZD7 to suppress FZD7 expression in GC (Geng et al., 2016). Double-stranded miR-30a is transformed to two single-stranded miRNAs, including miR-30a-3p and miR-30a-5p. The former regulates β-catenin nuclear translocation by inhibiting COX2, while the latter targets BCL9 to regulate TCF/LEF promoter activity. In *H. pylori*-infected GC, miR-30a plays a tumor suppressor role in cancer development (Liu et al., 2017). *H. pylori* infection in GC increases the level of miR-99b, which inhibits mTOR expression to upregulate autophagy, inducing intracellular *H. pylori* elimination and cell death (Yang L. et al., 2018).

However, there are also reports that miR-146 and miR-let-7 are significantly downregulated in *H. pylori*-infected GC (Ranjbar et al., 2018). MiR-146a acts as a tumor suppressor, as it reduces the expression of pro-metastatic genes like L1CAM and ROCK1 (Shomali et al., 2019). Some miRNAs have their own target genes and regulate the progression of GC at various time points. MiR-152 and miR-200b inhibit B7-H1 [a member of the B7 costimulatory family of molecules that bind to programmed death-1 (PD-1) and play a critical immunoregulatory role in the cell-mediated immune response] expression by binding to its 3′UTR, while *H. pylori* infection inhibits the ability of the miRNA to promote B7-H1 expression (Xie et al., 2017). *H. pylori* infection in GC tissue promotes miR-222-3p expression, decreasing the levels of its target HIPK2 (homeodomain-interacting protein kinase 2) and thus promoting proliferation and invasion and inhibiting apoptosis (Tan et al., 2018). Another analysis showed no significant difference in the expression of miR-222 between *H. pylori*-positive and *H. pylori*-negative GC tissues (Noormohammad et al., 2016). *H. pylori* infection leads to miR-328 downregulation and CD44v9 (CD44, variant 9) upregulation, and this upregulation can enhance reactive oxygen species resistance to prevent cell death (Ishimoto et al., 2015). Clinical statistical analysis showed that miR-375 downregulation and upregulation of its target JAK2 (Janus kinase 2) were associated with *H. pylori* infection in patients with GC (*p* < 0.05) (Chen B. et al., 2017). MiR-375 is regarded as an inhibitor of *H. pylori*-induced gastric carcinogenesis by inhibiting the expression of lncRNA SOX2OT (SOX2 overlapping transcript) and SOX2, a master regulator of the pluripotency of cancer stem cells (Shafiee et al., 2016). MiR-375 regulates the JAK2–STAT3 pathway, which affects BCL2 and TWIST1 expression to promote neoplastic transformation (Miao et al., 2014). A special miRNA requiring attention in *H. pylori* infection is the elevation of miR-30d expression, which enhances *H. pylori* intracellular survival via downregulation of the autophagy pathway (validated by several genes like ATG2B and BECN1) (Yang X. et al., 2016) (summarized in Table 2).

**TABLE 2:** Function of miRNA in Helicobacter pylori-infected gastric cancer.

MicroRNA (host gene)	Target gene of miRNA	Effector produced by H. pylori	Expression after H. pylori infection	Function to cancer after infection	References
MiR-34b			Down		Tahara et al., 2019
MiR-34c			Down		Tahara et al., 2019
MiR-124a	IL-8, SMOX		Down		Tahara et al., 2019
MiR-133a			Down		Hyun Lim et al., 2018
MiR-149	COX2, PGF2, IL-6		Down		Li et al., 2015
MiR-200a/b			Down		Choi et al., 2020
MiR-204(TRPM3)	BIRC2, NF-κB		Down	Metastasis, proliferation	Chen et al., 2019
MiR-210	STMN1, DIMT1	CagA	Down	Proliferation	Kiga et al., 2014
MiR-490-3p(CHRM2)	SMARCD1		Down	Viability, migration, invasion, colony formation, cell growth	Shen et al., 2015; Cho et al., 2016
let-7b	IL-1β, IL-8, Ras oncoprotein		Down	Immune response	Chen et al., 2015; Wu et al., 2017; Zhang et al., 2017
MiR-7	NF-κB, IKKε, RELA, FOS		Down	Proliferation	Zhao et al., 2015
MiR-22	NLRP3, IL-1β, CCND1		Down	Proliferation, inflammation	Li et al., 2018
MiR-24-3p			Down	Growth, migration, invasion, apoptosis	Li et al., 2016
MiR-30a-3p	β-Catenin, COX2		Down		Liu et al., 2017
MiR-30a-5p	BCL9, TCF/LEF		Down		Liu et al., 2017
MiR-34a	TLP4, TNF-α, IL-6	rTip-α	Down	Proliferation, viability	Wang et al., 2018
MiR-101/26	SOCS2, c-myc, CDK2, CDK4, CDK6, CCND2, CCND3, CCNE2; p14 p16, p21, p27		Down	Proliferation, colony formation	Zhou et al., 2015b
MiR-106b/375	JAK1, STAT3	LPS	Down		Ye et al., 2015
MiR-128/-148a	MMP-3/-7, E-cadherin		Down	Migration, invasion	Yang Y. et al., 2018
MiR-134	FoxM1	CagA, PBP1A	Down	Proliferation, invasion	Huang et al., 2019
MiR-145			Down		Demiryas et al., 2019
MiR-152, miR-200b	B7-H1(PDL1)		Down		Xie et al., 2017
MiR-204	SOX4		Down	Invasion, proliferation	Zhou et al., 2014a
MiR-320	Mcl-1	CagA	Down	Apoptosis	Noto et al., 2013
MiR-328	CD44v9		Down	Cell death	Ishimoto et al., 2015
MiR-375	SOX2OT, SOX2, JAK2–STAT3, BCL2, TWSIT1		Down	Cell proliferation, migration	Miao et al., 2014; Ye et al., 2015; Shafiee et al., 2016; Chen B. et al., 2017
MiR-490-3p			Down	Lymph node metastasis	Qu et al., 2017
MiR-1915	RAGE		Down	Proliferation, invasion, migration	Xu et al., 2019
MiR-3178	TRAF3, TNF-α and IL-6, NF-κB	Tip-α	Down	Inflammation	Zou et al., 2017
MiR-141	KEAP1		Down		Zhou et al., 2014b
MiR-143-3p	AKT2		Down		Wang F. et al., 2017
MiR-370	FoxM1	CagA	Down		Feng et al., 2013
MiR-21	RECK		Up		Zhang et al., 2008
MiR-30d	ATG2B, ATG5, ATG12, BECN1, BNIP3L		Up	Autophagy	Yang X. et al., 2016
MiR-99b	mTOR		Up	Autophagy, cell death	Yang L. et al., 2018
MiR-194			Up		Demiryas et al., 2019
MiR-146a	IRAK1, TRAF6, MyD88, TLRs, NF-κB, L1CAM, ROCK1		Up	Metastasis	Zabaglia et al., 2018; Li et al., 2019; Shomali et al., 2019
MiR-150-5p, miR-155-5p, and miR-3163	POLD3, MSH2, MSH3		Up	DNA damage, DNA repair	Santos et al., 2017
MiR-155	KLF4	CagA	Up	EMT, growth	Ou et al., 2019
MiR-221,222	RECK, PTEN		Up	Growth, invasion	Liu et al., 2015
MiR-223-3p	HIPK2, NF-κB, ARID1A, E-cadherin	CagA	Up	Proliferation, invasion, apoptosis	Ma et al., 2014; Tan et al., 2018; Yang F. et al., 2018- +
MiR-21			Up	EMT, inflammation	Zhu et al., 2019
MiR-135b-5p	NF-κB, KLF4		Up	Apoptosis	Shao et al., 2019
MiR-185	DNMT1, EZH2		Up		Yoon et al., 2013
MiR-223	FBXW7		Up		Zhou et al., 2015a
MiR-1289	HKα (H-K-ATPase α subunit)	CagA, SLT	Up	Transient hypochlorhydria	Zhang et al., 2014
MiR-223-3p			Up		Yang F. et al., 2018
MiR-29a-3p	A20		Up		Sun et al., 2018
MiR-320a, miR-4496	β-Catenin, ABCG2	CagA		Metastasis	Kang et al., 2016, 2017
MiR-490-3p	SMARCD1				Shen et al., 2015
MiR-155	Rheb			Autophagy, immune system response	Wu et al., 2016
MiR-29b-1-5p	PHLPP1, MMP2, MMP9				Datta et al., 2018

#### MicroRNAs Influence the Drug Resistance of *Helicobacter pylori*-Infected Gastric Cancer

Many miRNAs are also related to drug resistance and impact the treatment of GC. Early *H. pylori* eradication and aspirin use have been suggested to prevent development of the intestinal metaplasia in GC (Wang et al., 2019), while miR-21, 155, and 233 have been suggested to have a positive correlation with *H. pylori* infection to spasmolytic polypeptide-expressing metaplasia (SPEM) (Hyun Lim et al., 2018). *H. pylori* infection elevates miR-21 expression, while COE (*Celastrus orbiculatus*) inhibits this upregulation. COE upregulates PDCD4 expression by decreasing the methylation of its promoter and inhibits *H. pylori*-induced inflammation and EMT (Zhu et al., 2019). *H. pylori* infection enhances miR-135b-5p expression in a TNF-α-induced NF-κB-dependent manner and binds to KLF4 to attenuate its expression. The miRNA suppresses apoptosis and induces cisplatin resistance (Shao et al., 2019). *H. pylori* infection downregulates miR-141, thus reducing its target KEAP1, which enhances cisplatin resistance (Zhou et al., 2014b). GKN1 suppresses miR-185, which directly targets DNMT1 and EZH2 and exerts an anti-tumor effect together with 5-fluorouracil on tumor cell growth, while *H. pylori* infection causes GKN1 (Gastrokine 1) downregulation in GC cells (Yoon et al., 2013). *H. pylori*-infection elevates miR-223 expression, and it targets the 3′UTR of FBXW7 to modulate its expression and the G1/S transition of the cell cycle. Additionally, miR-223 shows cisplatin resistance, which can be reversed by overexpression of FBXW7 (Zhou et al., 2015a). Accompanied by *H. pylori* infection, CagA induces chemoresistance and CIC (cancer-initiating cell) properties like self-renewal and tumor-initiating capacity, while miR-320a and miR-4496 target β-catenin and ABCG2 (ATP-binding cassette, subfamily G, and member 2) at the transcriptional and posttranscriptional levels to attenuate CagA induction. Furthermore, the combination treatment of miR-320a/-4496 with 5-fluorouracil in an orthotopic mouse model has been shown to attenuate gastric tumorigenesis and metastatic potential (Kang et al., 2017). Rebamipide upregulates miR-320a/-4496 to suppress *H. pylori* CagA-induced β-catenin and CIC marker gene expression. This treatment could enhance sensitivity to chemotherapeutic drugs (Kang et al., 2016). It was found that the expression of miR-320a was downregulated in GC cells, and the sensitivity of GC cells to DDP was enhanced by directly regulating to ADAM10 (Ge et al., 2017). And miR-29b can enhance the sensitivity of GC cell by directly targeting PI3K/Akt pathway (Chen et al., 2015). In addition, the low expression of miR-125b (Zhang et al., 2017), miR-181a (Zhao et al., 2016), miR-22 (Qian et al., 2017), and so on was found to be associated with DDP resistance in GC. On the other hand, the development of MDR is also a key cause of treatment failure in GC, and it was found UCA could increase MDR of GC by directly downregulating miR-27b (Fang et al., 2016) (summarized in Table 3).

**TABLE 3:** Drug resistance-associated miRNA in Helicobacter pylori-infected gastric cancer.

MicroRNA	Associated medicine	References
MiR-124a	5-Azacytidine	Tahara et al., 2019
MiR-21, 155, and 233	Spasmolytic polypeptide	Kuo et al., 2019
MiR-21	*Celastrus orbiculatus*	Zhu et al., 2019
MiR-135b-5p	Cisplatin	Shao et al., 2019
MiR-141	Cisplatin	Zhou et al., 2014b
MiR-185	5-Fluorouracil	Yoon et al., 2013
MiR-223	Cisplatin	Zhou et al., 2015a
MiR-320a, miR-4496	5-Fluorouracil	Kang et al., 2016, 2017

Several miRNAs have been found to be biomarkers for the prognosis of GC. MiR-490-3p is downregulated in *H. pylori*-positive GC and is significantly correlated with lymph node metastasis and clinical stage (Qu et al., 2017). According to microarrays and RT-PCR, miR-145 is downregulated and miR-194 is upregulated significantly in *H. pylori*-positive GC (Demiryas et al., 2019). Urinary miR-6807-5p and miR-6856-5p perform as biomarkers when combined with *H. pylori* infection (ACU = 0.885) in the detection of GC (Iwasaki et al., 2019). The mechanism remains unclear. It may provide doctors with another way to quickly detect GC in the future.

## Conclusion

There are presently many reports on the mechanism of ncRNAs in relation to GC, but research on the mechanism related to *Helicobacter pylori* infection has not attracted sufficient attention. For instance, GClnc1 (GC-associated lncRNA 1) has been regarded as a modular scaffold of WDR45 and KAT2A histone modifiers. It can regulate the localization and histone modification of SOD2. GClnc1 shows a strong correlation with the carcinogenesis, invasion, growth, and prognosis of GC. Another is clinical analysis showed that 78% of GC patients with higher GClnc1 expression are *H. pylori*-infected. The mechanism underlying the interaction between *H. pylori* and GClnc1 merits further exploration (Sun et al., 2016).

Most current research mainly shows the correlation and interaction (promotion or interference) of ncRNA in *H. pylori*-infected GC. *H. pylori* infection in GC causes expression changes in miRNAs or lncRNAs, while miRNAs and lncRNAs can interact with each other. Another miRNA in turn affects the efficiency of *H. pylori* infection. Data analysis has mainly been derived from clinical data, databases, or sequencing or array analyses of *H. pylori*-infected normal gastric epithelial cells. In this case, a large number of research targets are obtained, but there remains a lack of further clarification of the specific molecular mechanism, which needs to be further confirmed and evaluated in animal models or *in vitro* experiments to provide more reliable evidence for clinical treatment. Concurrently, in analyses of clinical data, focusing on the correlation and interaction between ncRNA and various *H. pylori* virulence factors also provides a good perspective. Array and database analyses have provided large amounts of data, but they need to be further confirmed and analyzed, a stage of research that is still in the preliminary phases. NcRNA plays a very important role in the progression of *H. pylori*-infected GC. It can not only affect the chemotherapy resistance of GC but also serve as a biomarker for the prognosis of GC. As a new type of ncRNA, circRNA has been found to have the potential as a prognostic biomarker for GC (Shan et al., 2019). For example, the expression of hsa_circ_0001649 in GC was significantly lower than that in paired non-tumor tissues. What is more, compared with preoperative plasma samples, the expression level of hsa_circ_0001649 was upregulated after surgery, suggesting that hsa_circ_0001649 may be a follow-up indicator for GC patients after surgery (Li et al., 2017). And CircPVT1 levels were observed to be independent prognostic indicators of overall survival and DFS in GC patients (Chen J. et al., 2017). At present, ncRNAs including circRNAs, miRNAs, and lncRNAs have the potential to be used as prognostic biomarkers for GC, and previous studies have shown that many ncRNAs can accurately predict the prognosis of patients, which is of great significance to both doctors and patients. However, more work and efforts are still needed to be done for clinical application. Actually, we can found that ncRNA has been studied much longer than lncRNAs, and its experimental technique is more mature. And at the same time, there are more research results. But it could not prove that miRNA plays a more important role than lncRNAs in *H. pylori*-infected GC. The mechanism about lncRNAs still needs to be explored. And there are many kinds of ncRNAs in which their function has not been discovered in *H. pylori*-infected GC, and it would be a very promising research direction in the field of biomedicine.

## Author Contributions

CW and YH wrote the manuscript. SW, BZ, and YB revised the manuscript. YH and QL was responsible for searching the references. CY projected and edited the manuscript. XX and SY reviewed the manuscript. All authors read and approved the final manuscript.

## Conflict of Interest

The authors declare that the research was conducted in the absence of any commercial or financial relationships that could be construed as a potential conflict of interest.

## Abbreviations

ABCG2: ATP-binding cassette subfamily G member 2
CagA: cytotoxin-related gene A
cag-PAI: cag pathogenicity island
ceRNA: competing endogenous RNA
CHRM2: cholinergic receptor muscarinic 2
CIC: cancer-initiating cell
circRNAs: circular RNAs
EMT: epithelial–mesenchymal transition
FoxM1: Forkhead box protein M1
FZD7: Frizzled 7
GC: gastric cancer
GClnc1: GC-associated lncRNA 1
GKN1: Gastrokine 1
GML: gastric MALT lymphoma
*H. pylori*: *Helicopter pylori*
HIPK2: homeodomain-interacting protein kinase 2
IRAK1: IL-1 receptor-associated kinase 1
JAK2: Janus kinase 2
KLF4: Krüppel-like transcription factor
lncRNAs: long non-coding RNAs
LPS: lipopolysaccharide
miRNAs: microRNAs
MMR: mismatch repair
PBP1A: penicillin-binding protein 1A
piRNAs: PiWi-interacting RNAs
PRNCR1: prostate cancer non-coding RNA 1
RAGE: receptor for advanced glycation end product
RepG: regulator of polymeric G-repeats
siRNAs: small interfering RNAs
SMARCD1: SWI/SNF chromatin remodeling complex subunit
SMOX: spermine oxidase
snoRNAs: small nucleolar RNAs
SOX2OT: SOX2 overlapping transcript
SSRs: simple sequence repeats
SWI/SNF: SWItch/Sucrose Non-fermentable
TCS: two-component system
THAP9-AS1: THAP domain-containing 9 antisense RNA 1
TRAF6: tumor necrosis factor receptor-associated factor 6
tsRNAs: tRNA-derived small RNAs
ZEB1: zinc-finger E-box -binding homeobox 1
ZEB2: zinc finger E-box binding homeobox 2.
